# Complete Genome Sequence of a Novel Iflavirus from the Transcriptome of *Halyomorpha halys*, the Brown Marmorated Stink Bug

**DOI:** 10.1128/genomeA.00910-13

**Published:** 2013-11-27

**Authors:** Michael E. Sparks, Dawn E. Gundersen-Rindal, Robert L. Harrison

**Affiliations:** USDA-ARS Invasive Insect Biocontrol and Behavior Laboratory, BARC-West, Beltsville, Maryland, USA

## Abstract

We report the genome sequence of a novel iflavirus recovered from the transcriptome of a colony of the brown marmorated stink bug, *Halyomorpha halys*. The genome is 9,263 nucleotides (nt) and contains an open reading frame (ORF) encoding a 3,017-amino-acid polyprotein.

## GENOME ANNOUNCEMENT

The brown marmorated stink bug (BMSB), *Halyomorpha halys* (Heteroptera: Pentatomidae), is native to China, Japan, Korea, and Taiwan. Since its appearance near Allentown, PA, in the mid-1990s, it has spread to 38 states in the United States (1). Beginning in 2010, heavy infestations of BMSB have caused millions of dollars of fruit and vegetable crop losses in the U.S. states of MD, WV, VA, and PA. Methods for monitoring and managing this pest are being pursued with great urgency.

As part of a transcriptomic study of *H. halys*, BMSB nymphs and adults from a USDA-ARS colony at Beltsville, MD, were homogenized with an Ultra-Turrax T25 (IKA Labortechnik), and RNA was extracted using the mirVana miRNA isolation kit (Life Technologies). RNA sequencing (RNA-seq) libraries were prepared using the mRNA-Seq sample prep kit (Illumina) and sequenced using an Illumina GAII instrument. Approximately 196 million single-ended 100-bp BMSB RNA-seq reads were generated, of which approximately 113 million reads survived quality control. These reads were assembled into putatively unique transcripts using Trinity (2). BLASTx comparisons with the NCBI NR protein database revealed a 9,264-nucleotide (nt) contig in the transcriptome that shares approximately 70% nucleotide sequence identity with a partial sequence of an iflavirus, strain P1/InV1/IT/USA/2009 (3). This transcript terminates in a 3′-polyadenylated tail and has a G+C content of 37.33%. It contains a 9,051-nt open reading frame (ORF) with a 152-nt 5′-untranslated region (UTR) and a 61-nt 3′-UTR. The presence of this transcript in BMSB RNA was confirmed by PCR and dideoxy sequencing of cDNA prepared with SuperScript II (Invitrogen) using overlapping sets of primers. Rapid amplification of cDNA ends (RACE) using the SMARTer RACE cDNA amplification kit (Clontech) determined that this transcript contains the sequence of the entire genome, with 5′-RACE indicating that the nucleotide closest to the 5′-end of the contig assembly was not present in the genome.

The 3,017-amino-acid sequence specified by the ORF revealed a polyprotein with a domain arrangement similar to that described for other iflaviruses (4). Three rhv-like (Pfam entry, cd00205; picornavirus capsid protein domain-like) motifs were identified at amino acid positions 312 to 453, 491 to 678, and 833 to 989, while RNA helicase (Pfam entry, PF00910) and RNA-dependent RNA polymerase (RdRp) (Pfam entry, cd01699) domains were found at positions 1482 to 1575 and 2646 to 2941, respectively. BLASTp with the RdRp domain amino acid sequence produced the highest-quality matches with P1/InV1/IT/USA/2009, sacbrood virus (5), and *Lygus lineolaris* virus 1 (6), with amino acid sequence identities of 82.4%, 47.7%, and 38.9%, respectively.

We conclude that the sequence reported here represents the complete genome sequence of a previously undescribed iflavirus that resides in BMSB. Iflaviruses and other insect picorna-like viruses generally establish chronic asymptomatic infections of their hosts. One notable exception is the *Solenopsis invicta* (fire ant) SINV-3, which was observed to cause significant mortality in fire ant colonies when worker ants were fed the virus (7). Further research with the BMSB virus is planned to determine its pathogenicity and pest control potential.

### Nucleotide sequence accession number.

The genome sequence was deposited in GenBank under the accession no. KF699344.
